# 

**Published:** 1952-04

**Authors:** 


### Contents

| | | Page |
|---|---|---|
| EDITORIAL | | 33 |
| THE PREVENTION AND TREATMENT OF POSTPARTUM HAEMORRHAGE IN DOMICILIARY PRACTICE | *Dame Louise McIlroy* | 35 |
| THE SYMPTOMS AND CLINICAL DIAGNOSIS OF HIATUS HERNIA | *Mr. Ronald Belsey* | 39 |
| CONTACT DERMATITIS | *Dr. Clifford Evans* | 45 |
| BRISTOL'S NEW HEALTH CENTRE | *Dr. R. C. Wofinden* | 49 |
| ON REACTIONS WITH THE NEWER ANTIBIOTICS | *Dr. P. G. Dalgleish* | 54 |
| VIEWS ANCIENT AND MODERN ON PLACENTA PRAEVIA | *Mr. John Stallworthy* | 57 |
| UNEXPLAINED PYREXIA IN A CHILD | CLINICO-PATHOLOGICAL REPORT | 60 |
| REPORTS OF MEETINGS | | 64 |

CLINICAL SOCIETY OF BATH:
- THE MEDICAL HAZARDS CONFRONTING THE MODERN AVIATOR

DEVON AND EXETER MEDICO-CHIRURGICAL SOCIETY:
- TONSILS AND ADENOIDS: DOGMAS AND DOUBTS
- THE SCOPE IN CLINICAL ELECTROCARDIOGRAPHY
- WHAT IS NEW IN ANAESTHESIA
- THE PROBLEMS OF GERIATRICS

SOUTH WEST ORTHOPAEDIC CLUB
- AUTUMN MEETING

WATSON-WILLIAMS MEMORIAL LECTURE
- DEFENCE OF THE AIR PASSAGES

| | | |
|---|---|---|
| ANNOTATIONS | | 69 |

- PROBLEM FAMILIES IN BRISTOL
- SOUTH WEST HOSPITALS' FINANCE IN 1952–53
- FAMILY PLANNING ASSOCIATION

| | | |
|---|---|---|
| CORRESPONDENCE | | 72 |

- MATERNITY SERVICE IN BRISTOL. Dr. O. E. L. Sampson; Dr. F. G. Mogg; Dr. A. G. Heron

| | | |
|---|---|---|
| APPOINTMENTS | | 74 |
| NOTES AND NEWS | | 74 |

